# A novel combinatorial treatment option for metastatic uveal melanoma

**DOI:** 10.18632/oncotarget.25445

**Published:** 2018-05-25

**Authors:** Dudi Shneor, Shay Tayeb, Jacob Pe'er, Hanna Voropaev, Maria Gimmelshein, Nathalie Cassoux, Alik Honigman, Shahar Frenkel

**Affiliations:** ^1^ Department of Biochemistry and Molecular Biology, IMRIC, The Hebrew University-Hadassah Medical School, Jerusalem, Israel; ^2^ Department of Ophthalmology, Hadassah-Hebrew University Medical Center, Jerusalem, Israel; ^3^ Hadassah Academic College, Jerusalem, Israel; ^4^ Department of Ocular Oncology, Institut Curie, Paris, France; ^5^ Université Paris V Descartes, Paris, France

**Keywords:** cancer, chemotherapy, replicative competent retroviruses (RCR), CREB, combinatorial targeted treatment

## Abstract

Uveal melanoma (UM) is the most frequent intraocular tumor in adult patients. When metastases occur, systemic therapy with alkylating agents (fotemustine or dacarbazine (DTIC)) has shown only modest efficacy. The common chemotherapeutic drug doxorubicin (DOX) is not used to treat metastatic UM (mUM). To expand the chemotherapeutic arsenal for mUM, we tested the effect of DOX on UM cell mortality. We have previously shown that CREB knockdown enhances sensitivity to DOX. UM cells infected with recombinant MuLV-based replicative competent retroviruses (RCR) expressing shRNA targeting CREB were co-treated with either DTIC or DOX. We found that CREB knockdown increases the sensitivity of these cells to both DOX and DTIC in normoxia and more so in hypoxia as measured by cell survival and Caspase 3 activation. The ability to combine CREB knockdown by infection with the RCR recombinant virus which preferentially infects replicating tumor cells and chemotherapy to achieve the same amount of cell death in lower concentrations may result in fewer side effects of the drugs. This combination is a possible new treatment for mUM.

## INTRODUCTION

Uveal melanoma (UM) is the most frequent intraocular tumor in adult patients. Up to 50% of patients will develop metastases [1], of which 80% die in the first year, and 92% within the first two years [2]. Systemic therapy with alkylating agents, i.e., fotemustine or dacarbazine (DTIC), have shown only modest efficacy [3, 4]. Consequently, because of the limited efficacy of current treatments, new therapeutic strategies need to be developed.

One of the primary means by which UM cells evade treatment-induced apoptosis is by up-regulation of members of the pro-survival Bcl-2 family proteins such as Bcl-2 and Bcl-X_L_ [5, 6]. Indeed, up to 82% (range: 44%–100%) of human UMs are characterized by elevated Bcl-2 levels [7–11] without any prognostic impact [7, 10–12].

The transcription factor cyclic AMP-response-element (CRE) binding protein (CREB) was previously found by our team to suppress Hepatocellular Carcinoma (HCC) cell death under hypoxic conditions. Overexpression of positive dominant CREB300/310 prevented cell death in hypoxia [13]. On the other hand, CREB knockdown increased HCC cell sensitivity to hypoxia as well as to doxorubicin (DOX) in normoxia and hypoxia [14]. Recently, it was demonstrated that CREB blockade by decoy oligonucleotides functionally inhibited transactivation of CREB, and significantly increased radio-sensitivity of multiple human cancer cell lines [15]. Overexpression of CREB decreases expression of the pro-death Bim protein and inhibits the sequestration of Bim protein from tubulin molecules, thereby protecting cells from apoptosis [16]. Additionally, activation of Bcl-2 expression by CREB promotes cell survival, while Trichosanthin's inhibition of CREB's activation of cell cycle regulatory proteins such as cyclin A resulted in cell cycle arrest [17]. CREB is also involved in the apoptotic effect of DOX, where DOX-induced p38 activation can suppress the PKA pathway, preventing activation of CREB and thus leading to downregulation of Bcl-2 and as a result to enhanced apoptosis [18]. Sayan et al found that combining inhibition of CREB phosphorylation with DOX treatment was significantly more effective in mesotheliomas [19]. These findings along with our ability to increase tumor cell sensitivity to DOX led us to test if this combination is also effective in UM.

Although DOX is a well-accepted chemotherapeutic agent in a variety of metastatic neoplastic diseases [20], it is hardly used for metastatic uveal melanoma. An extensive literature review identified only a single case report by Brasiuniene et al. that describes the use of DOX in combination with two other chemotherapies and interferon α for metastatic UM with liver lesions after other treatments have failed. This therapy led to a partial response and was later followed by resection of the metastases [21]. Recently, Latorre and colleagues used gold nanoparticles (GNPs) for targeted delivery of a high concentration of DOX to UM cell lines. They demonstrated that modified GNPs could be functionalized to increase the efficacy of cancer therapeutics and may further reduce toxicity by increasing targeted delivery towards malignant cells [22].

In this work, we infected the cells with a MuLV-based recombinant replication competent retrovirus (RCR) [23] which expresses shRNA targeting CREB (vACE-CREB) [14] prior to treatment of the tumors with chemotherapeutic drugs. The advantage of using this vector is that MuLV retroviruses infect only replicating cells such as tumor cells, generating a stably integrated provirus in the cells. These cells produce viral particles expressing shRNA targeting CREB that will spread within the tumor [14].

Solid tumors grow faster than they can attract blood vessels into them generating an ongoing formation of hypoxic regions within tumors. In these regions, tumor cells are too far from the present vessels, and thus oxygen and drugs do not reach them. This poses a problem in killing solid tumors: these areas are radio-resistant due to the lack of active oxygen radicals, and are chemo-resistant due to the lack of tumoricidal concentrations of the drugs [24]. Unlike normal tissues, solid tumors are more resistant to hypoxia. CREB was found to play a pivotal role in the response of cells to hypoxia [13] along with the hypoxia-inducible factors 1 and 2 (HIF-1 and HIF-2). To overcome the problem of these resistant regions to chemotherapeutic treatments, we constructed additional RCR vectors expressing shRNA targeting the three hypoxia-response regulating genes. We have shown a decreased survival in hypoxia of the recombinant RCR infected HCC cells and an increase in the sensitivity of these infected cells in hypoxia to DOX [14].

Targeting the unique property of ongoing generation of hypoxic areas in tumors by the RCRs along with the preference for replicating cells, will preferentially affect tumors in a double-targeting mechanism. We demonstrate here that infecting UM cells with vACE-CREB sensitizes them to treatment with DOX and DTIC.

The increased sensitivity to chemotherapy in the RCR-infected cells *in vitro* may set the basis for a more efficient combined treatment for metastatic UM.

## RESULTS

### Infectivity of the recombinant RCRs in UM cell lines

**Figure 1:**
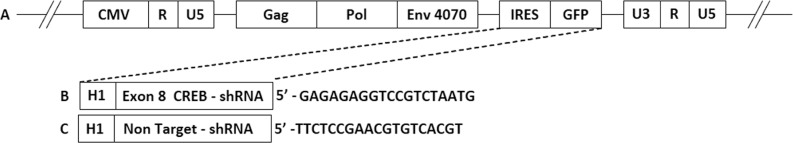
A schematic presentation of the various RCRs **(A)** The provirus construct of pACE-GFP. **(B, C)** Replacement of the IRES-GFP sequences with an H1 promoter driving the transcription of shRNAs. Sequences coding for the shRNA are specified.

To construct a MuLV replicating viral vector that expresses shRNA targeting CREB (Figure 1) the IRES-GFP DNA fragment in vACE-GFP [23] was replaced by the H1 promoter driving the shRNA sequences targeting CREB (pACE-CREB) or expressing a non-target sequence (pACE-NT) as previously described [14].

The titer of the viral preparations was defined by comparison of qPCR of the *env* gene to RNase P (a single copy gene per cell) in cells 48 hours after infection. This method quantifies the infective particles in the viral preparations.

**Figure 2:**
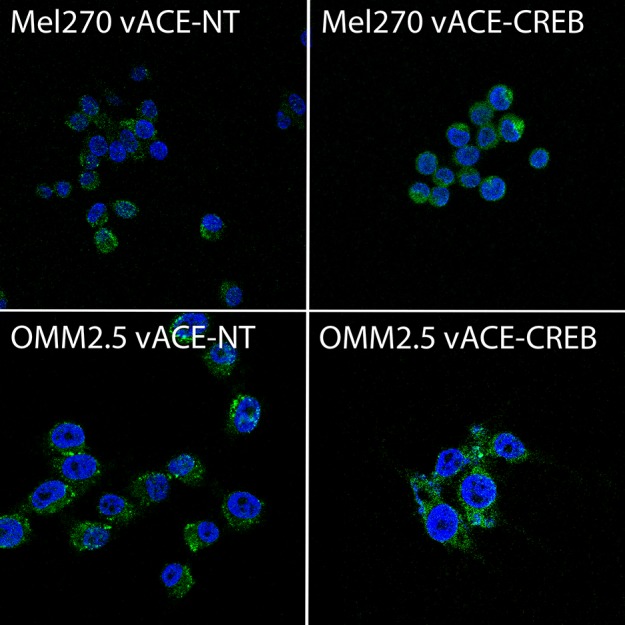
Immunofluorescence assessment of recombinant RCR infectivity For each slide, Hoechst labeled nuclei (blue) were counted. The staining of viral particles in the cytoplasm of these cells (green) was recorded (x63 magnification). All cells show about a 90% ratio of green- to blue-labeled cells.

GFP fluorescence from cells infected with vACE-GFP served to determine the kinetics of spread of the virus in Mel 270 and OMM2.5 cells in culture. The efficiency of infectivity was verified by immunofluorescent staining of the vACE-CREB and vACE-NT infected cells. It takes about three weeks for GFP fluorescence to indicate that about a 100% of the vACE-GFP cells were infected. At the same time, immunofluorescence analyses (Figure 2) and qPCR ratio of the viral *env* gene vs. the endogenous RNaseP (not shown) in cells infected with either RCR showed that about 90% of the cells were infected with either vACE-NT or vACE-CREB.

### Knockdown efficiency

**Figure 3:**
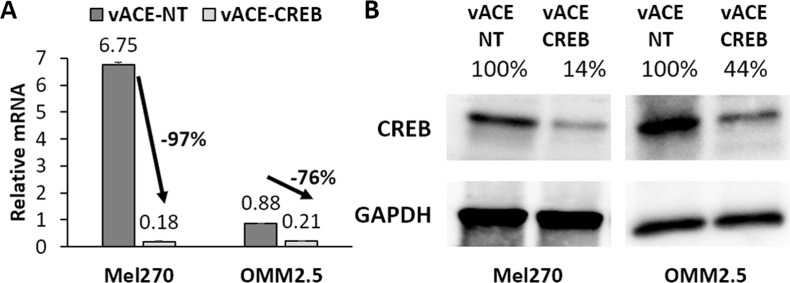
Quantification of the efficiency of knockdown in Mel 270 and OMM2.5 infected cell The knockdown of CREB in cells fully infected with either vACE-NT or vACE-CREB were analyzed for mRNA and protein levels. **(A)** Purified mRNA was quantified following RT-qPCR. mRNA levels were normalized to β-actin mRNA levels in the cells. A knockdown of CREB of 97% and 76% was noted in Mel270 and OMM2.5, respectively, p<0.05 (This is a summary of 4 repeats). **(B)** Western blot analysis of CREB protein (43 kDa). Protein band intensities were compared to those of GAPDH (37 kDa) and to the band intensity of cells infected with vACE-NT (set as 100%).

The efficiency of knockdown of CREB in vACE-CREB infected cells was determined by RT-qPCR and Western blot analyzes relative to cells infected with vACE-NT. Infection with vACE-NT did not change the expression of CREB mRNA and CREB protein significantly relative to the non-infected cells (data not shown) proving that the infection with the retrovirus did not affect the levels of CREB in the infected cells. Therefore, knockdown efficiency by vACE-CREB was compared to cells infected with vACE-NT. Baseline CREB mRNA levels greatly differed between the two cell lines with a 7.6 fold more CREB mRNA in Mel270 cells compared to OMM2.5 cells. Regardless of the initial level of CREB, vACE-CREB knocked down CREB mRNA levels in Mel270 and OMM2.5 to a similar low level (0.18 and 0.21, respectively) representing a knockdown of 97.4% and 76.1%, respectively (Figure 3A). The CREB protein levels decreased by 86% and 56% in Mel270 cells and OMM2.5 cells, respectively (Figure 3B). The minor differences in knockdown efficiencies between the two cell lines may represent differences in the expression of the shRNA and may depend on the initial levels of the target mRNA.

The effect of knockdown on the activity of CREB in the cells was monitored with a luciferase reporter gene plasmid and by measuring the expression of downstream endogenous genes.

**Figure 4:**
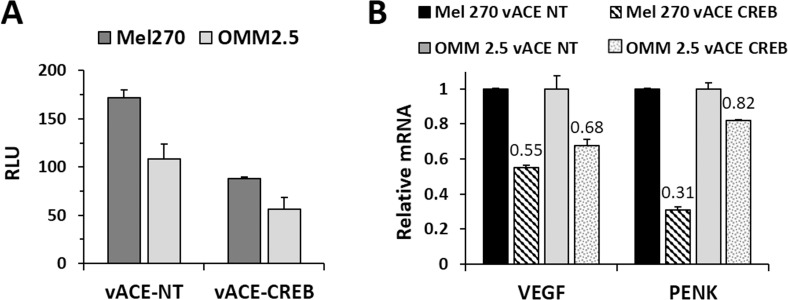
Functional analysis of the effect of CREB knockdown in Mel 270 and OMM2.5 infected cell The effect on the activity of CREB in the RCR-infected cells was determined in two ways: luciferase reporter gene and knockdown of downstream genes. **(A)** The infected cells were co-transfected with a CRE-mediated luciferase (luc) reporter plasmid vector, pCREluc together with an expression vector expressing the Renilla luciferase gene, phRLSV40, as a transfection control. The results were normalized to Renilla luciferase activity. **(B)** Purified mRNA was quantified following RT-qPCR. The levels of mRNA of VEGF and PENK in vACE-CREB infected cells were normalized to β-actin mRNA levels and to the levels of VEGF and PENK in vACE-NT infected cells, p<0.05.

Stably infected cells were transfected with a CRE-mediated luciferase gene expression reporter plasmid, pCREluc. Luciferase activity was determined 48h post-transfection. As expected from the initial CREB levels (Figure 3A), the luciferase activity in vACE-NT-infected OMM2.5 cells was 63% lower than that of Mel270 cells (Figure 4A). Knockdown of CREB resulted in a 36% reduction of luciferase activity in both cell lines (Figure 4). This result is in correlation with the similar residual levels of CREB in both cell lines (0.18 and 0.21, see above).

Additionally, we measured the expression of the endogenous genes vascular endothelial growth factor (VEGF) and that of proenkephalin (PENK). Knockdown of CREB in Mel270 cells resulted in a 45% and 32% reduction of VEGF and PENK mRNAs, respectively, and in a 69% and 18%, respectively, in OMM2.5 cells (Figure 4B).

### Effect of hypoxia and of chemotherapeutic drugs on apoptosis and cell viability

**Figure 5:**
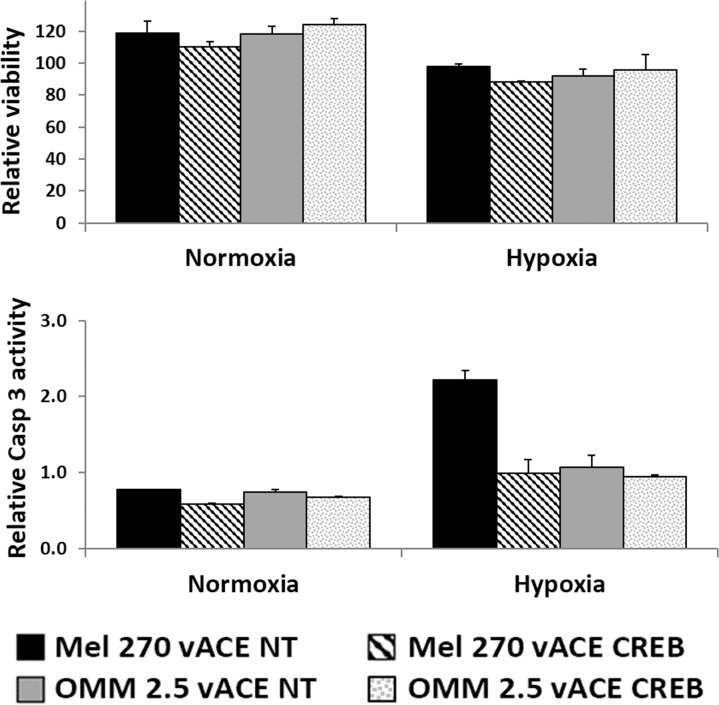
The effect of CREB on uveal melanoma's sensitivity to hypoxia Mel270 and OMM2.5 cell lines infected with either vACE-NT or vACE-CREB were cultivated in normoxia and hypoxia for 48 hours before the viability (top) and the activation of Caspase 3 (bottom) were determined by the Fluorescent Cell Viability and Caspase-Glo 3/7 Assay (Promega) (expressed in relative light units). There was no significant difference between the viability measurements, and the activation of Caspase 3 measurements were within the background levels.

The effect of CREB-knockdown on apoptosis in Mel270 and OMM 2.5 cells was determined. At normoxic conditions, CREB knockdown did not increase the apoptotic (sub-G1) fraction (flow cytometry, Table 1, lines 1 and 2) and barely affected cell viability and Caspase 3 activity (cell viability and activation of the Caspase-3 assay, Figure 5).

**Table 1:** The effect of CREB knockdown, treatment with DOX or DTIC and in combination with apoptosis in normoxia and hypoxia

	Cell lines	Normoxia			Hypoxia	
		Mel270	Omm2.5		Mel270	Omm2.5
1	No treatment (shNT)	1	1	Hypoxia/Normoxia	1	1
2	shCREB	NC	NC	Hypoxia/ NT in Hypoxia	1.6	1.5
3	DOX 0.5 μg	NC	1.5		1.2	NC
4	DOX 1 μg	NC	4.0		1.7	8.5
5	shCREB DOX 0.5 μg	2.2	1.2		2.3	5.1
6	shCREB DOX 1 μg	2.8	2.1		2.3	22.0
7	DTIC 400 μg	NC	NC		2.6	1.2
8	DTIC 600 μg	1.4	NC		7.7	2.2
9	shCREB DTIC 400 μg	1.3	NC		3.8	NC
10	shCREB DTIC 600 μg	4.6	2.5		9.5	9.8

The subG1 fraction (apoptotic cells) was defined by FACS analysis. The percent of apoptotic cells in hypoxia (48 Hr) in control, cells infected with vACE-NT (Line 1), was calculated relative to apoptotic cells in normoxia. The effect of the different treatments on induction of apoptosis was calculated relative to non-treated cells in normoxia or hypoxia which was set as 100%. NC=No change.

**Figure 6:**
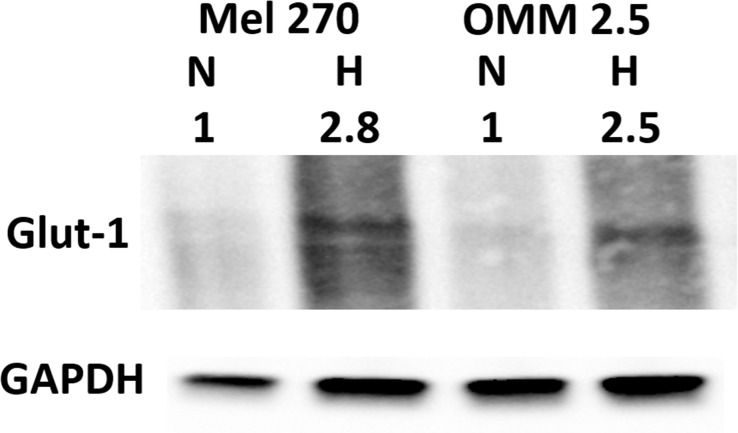
Cellular response to hypoxia Mel270 and OMM2.5 cell lines were cultivated in normoxic (N) and hypoxic (H) conditions for 48 hours and the relative expression of Glut-1 (50 kDa) was determined by Western blot analysis. Band intensities were normalized to GAPDH (37 kDa), and the intensities of hypoxia were normalized to those in normoxia.

Before assessing the effect of knockdown of CREB in hypoxia, we verified that the cells respond to the hypoxia, by increasing the expression of Glut-1. Indeed, following 48 hours of hypoxia, there is 2.5-2.8 fold increase in Glut-1 (Figure 6).

In hypoxia, CREB knockdown increased the apoptotic (sub-G1) fraction in both tested cell lines by about 50% (Table 1, lines 1 and 2).

In the cell viability and Caspase 3 assay, in normoxia, the cell lines grew by 17% on average after 48 hours. Hypoxia of 48 hours inhibited cell growth and barely increased Caspase 3 irrespective of the cell line or CREB level in these cells (note that the activate Caspase 3 measurements were within the background levels compared to the values in Figures 8 and 9). Thus, it seems that unlike HCC cells [14] the two UM cell lines are resistant *in vitro* to short hypoxia cues (48 hours), and can survive in either normoxia or hypoxia even with highly diminished levels of CREB.

**Figure 7:**
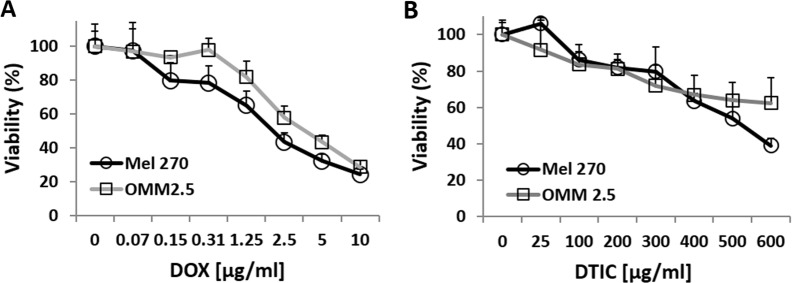
Dose response to treatment of UM cells with DOX or DTIC Mel270 and OMM2.5 cell lines were cultivated in normoxic conditions and treated with various concentrations of either DOX **(A)** or DTIC **(B)** for 48 hours and viability of the cells was determined by the Fluorescent Cell Viability Assay (Promega).

To calibrate the effective concentration of the chemotherapeutic drugs (DOX or DTIC), Mel270 and OMM2.5 were cultured for 24 hours with increasing doses of either drug. A dose-response curve of viable cells post-treatment was plotted to show that both cell lines were sensitive to both chemotherapeutic agents, albeit at different concentrations (Figure 7). These results served us to determine the suboptimal concentration (LD50) of DOX and DTIC that would allow detection of a synergistic effect when we test these chemotherapeutic agents on cells infected with vACE-CREB.

**Figure 8:**
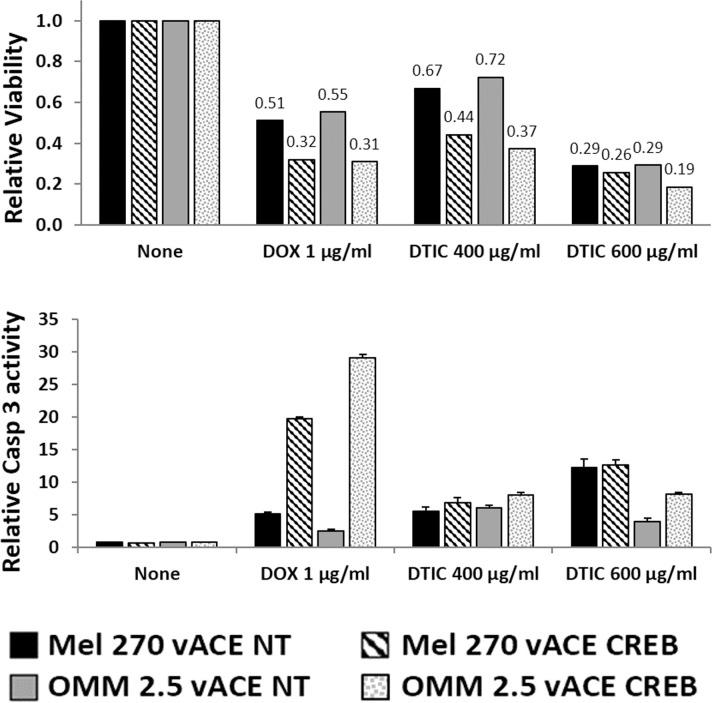
The effect of CREB on uveal melanoma's sensitivity to chemotherapy in normoxia Mel270 and OMM2.5 cell lines infected with either vACE-NT or vACE-CREB were cultivated in normoxic conditions and treated with either DOX (1μg/ml) or DTIC (400μg/ml or 600μg/ml) for 48 hours and viability (top) and activation of Caspase 3 (bottom), were determined relative to non-treated vACE-NT and vACE-CREB infected cells (set as 100% for each infected cell).

The effect of the selected chemotherapeutic concentrations (DOX 0.5 and 1 μg/ml, DTIC 400, 600 μg/ml) on induction of apoptosis in Mel270 and OMM 2.5 cells is presented in Table 1 (lines 3 and 4 - DOX, lines 7 and 8 – DTIC). In normoxia, 1 μg/ml of DOX barely increased the apoptotic fraction of Mel270, but in hypoxia, there was a 70% increase in the apoptotic fraction relative to non-treated cells in hypoxia. These results correlate with the moderate increase in Casp3 in Mel270 cells treated with 1 μg/ml Dox although about 50% of the cells died at this concentration of DOX (Figure 8). The apoptotic fraction in OMM2.5 increased by about 4-fold and 8-fold in response to 1 μg/ml of DOX in normoxia and hypoxia, respectively, indicating that OMM2.5 cells are more sensitive than Mel270 to DOX in normoxia and even more so in hypoxia. DTIC barely affected either cell line in normoxia, but there were more than 7- and 2-fold increases in the apoptotic fraction in treated Mel270 and OMM2.5 cells in hypoxia, respectively (Table 1, lines 7-8).

### Effectivity of combining treatment with either DOX or DTIC with vACE-CREB

The combined effect of DOX and CREB-knockdown on induction of apoptosis was determined by flow cytometry (Table 1, lines 5-6) where Mel270 cells had a two-fold increase in apoptosis in either normoxia or hypoxia. OMM2.5 also responded with a doubled apoptotic (sub-G1) fraction to treatment with 1 μg/ml concentration of DOX in normoxia, while there was a 22-fold increase in the apoptotic fraction to a concentration of 1 μg/ml of DOX in hypoxia.

In the complimentary viability and activation of the Caspase-3 assay, cells not treated with either DOX or DTIC and harboring stably either vACE-NT or vACE-CREB were defined as 100% (non-treated cells) (Figure 8).

We used the higher dose of DOX for testing the survival and activation of Caspase 3 (Figure 8) and found an about 20% additive reduction in survival and a 14-26-fold increase in activation of Caspase 3 in vACE-CREB infected cells vs. vACE-NT infected cells. Thus, the results obtained by the two independent methods correlate with each other.

DTIC is currently used to treat metastatic UM and has not been related directly to CREB activity. We used DTIC to test whether the ability of CREB knockdown to increase the sensitivity of cells to DOX can be generalized to other chemotherapeutic agents. To test if knockdown of CREB increases the cellular sensitivity to DTIC, we used two sub-lethal concentrations of DTIC (400 and 600 μg/ml). Treatment of vACE-CREB infected Mel270 cells (for 48 hours) with 400 μg/ml hardly affected their apoptotic fraction in normoxia with an almost 4-fold increase in hypoxia. However, use of 600 μg/ml resulted in about 4.5-fold increase in the apoptotic (sub-G1) fraction in normoxic conditions and a 9.5-fold increase in hypoxic conditions (Table 1, lines 9-10). OMM2.5 cells were less sensitive than Mel270 cells to treatment with DTIC even in reduced CREB conditions. vACE-CREB infected OMM2.5 cells did not respond to 400 μg/ml of DTIC in either normoxia or hypoxia. However, there was a two-fold increase in apoptosis with 600 μg/ml of DTIC in normoxia and a 9.8-fold increase in apoptosis in hypoxia.

**Figure 9:**
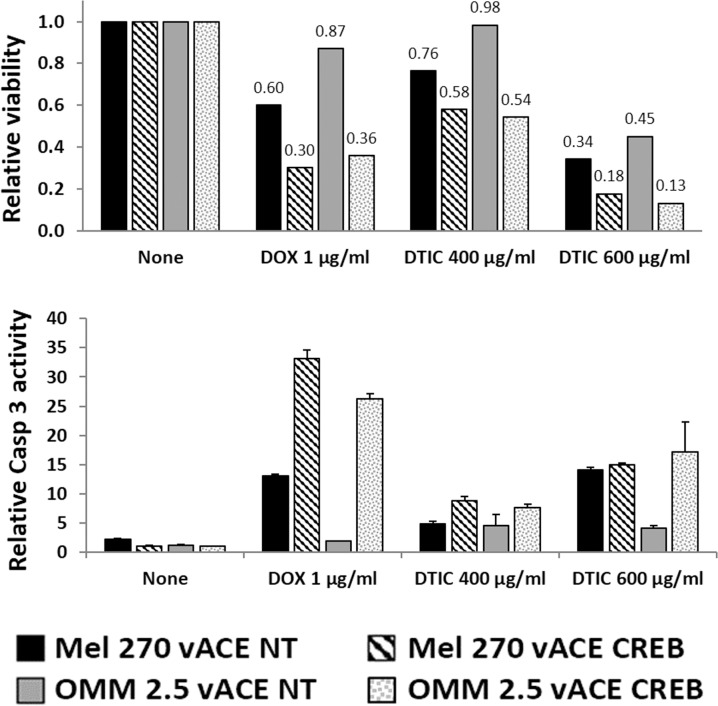
The effect of CREB on uveal melanoma's sensitivity to chemotherapy in hypoxia Mel270 and OMM2.5 cell lines infected with either vACE-NT or vACE-CREB were cultivated in hypoxic conditions and treated with either DOX (1μg/ml) or DTIC (400μg/ml or 600μg/ml) for 48 hours. Viability (top) and activation of Caspase 3 (bottom), were normalized to non-treated vACE-NT and vACE-CREB infected cells (set as 100% for each infected cell) and expressed in relative light units.

The complementary analysis of survival and Caspase 3 activation showed an additive 20-40% reduction in survival with 400 μg/ml and a 16-32% additive reduction with 600 μg/ml (where the drug itself is more toxic) with a parallel additive increase in the activation of Caspase 3 (Figure 9). Comparison of the killing effect of DTIC on vACE-NT infected cells in hypoxia (Figure 9) vs. normoxia (Figure 8) shows that DTIC is less effective in hypoxia. Taking into account the above findings that these UM cells are insensitive to hypoxia, the additive killing noted in the vACE-CREB infected cells in hypoxia suggests that CREB activity protects cells from DTIC in hypoxia.

## DISCUSSION

Solid tumors grow faster than they can attract blood vessels into them generating an ongoing formation of hypoxic regions within tumors. In these regions, tumor cells are too far from the present vessels, and thus oxygen and drugs do not reach them. In previous work, we have shown that knockdown of CREB prevents the cellular responses to hypoxia in hepatocellular carcinoma, increase cell death in hypoxia, and lead to increased sensitivity to treatment with doxorubicin in normoxia and hypoxia, *in vitro* and *in vivo* [14].

Based on these results we investigated whether infective knockdown of CREB by the retroviral replication competent vector will be effective on other tumors.

Metastatic uveal melanoma remains a therapeutic challenge with no effective chemotherapeutic or biologic treatment. In this work, we present a novel combinatorial system that may open an avenue for effective treatment for mUM.

As illustrated in Table 1 (line 1) and Figure 5, uveal melanoma cell lines are insensitive to hypoxia. Since we demonstrated that CREB plays a pivotal role in the cellular responses to hypoxia, we knocked down CREB in two UM cell lines and found a 50% increase in the apoptotic fraction following knockdown of CREB (Table 1, line 2). Thus, CREB is involved in the resistance to hypoxia cue in these cell lines, although the increase in the apoptotic fraction was not expressed in cell death (Figure 5). This discrepancy may result from the different sensitivities of flow cytometry (apoptotic fraction) vs. the Fluorescent Cell Viability and Caspase-Glo 3/7 Assay. These results may suggest that Caspase 3 is not involved in the generation of the sub-G1 fraction. Additionally, the gap between the increase in apoptosis and the lack of increase in cell death reflects the higher resistance of UM cells to hypoxia relative to HCC cells.

Knockdown of CREB resulted in a decrease in the expression of VEGF in the UM cells (Figure 4), similar to our findings in HCC [14], which could contribute to a decrease in tumor cell growth *in vivo* due to inhibition of perfusion of the growing tumors and increase the hypoxic areas within the tumors. Thus, infection with vACE-CREB will, on the one hand, increase the hypoxic regions, and on the other hand, prevent the tumor cells from responding to the hypoxia cue.

Cell viability in response to chemotherapy of 77 choroidal melanomas and 58 cutaneous melanomas was assessed using an *ex vivo* ATP-based chemosensitivity assay (ATP-TCA) [4]. Of 12 different chemotherapeutic agents and one of two combinations, almost none of the UMs responded to the chemotherapy. DTIC is in use in some centers to treat mUM with a limited success rate [25, 26]. DOX, has been used to treat primary liver tumors, which has not been used for mUM in which almost a 100% of the patients present with liver metastases. In this work we found that two separate UM cell lines responded to these two agents, DTIC and DOX (Figures 8 and 9), albeit not equally so.

There could be several explanations for the effect we found versus Naele's results. One would be that we used different concentrations of the drugs. Another explanation is that these cell lines represent the outliers that do respond to chemotherapy. The world of oncology is moving in the direction of personalized medicine as we learn more about inter-patient variations, inter-tumor variability, and how different patients with what was thought of as the same tumor respond differently to chemotherapy [27]. The implication is that outliers should not be ignored and that these two cell lines may represent at least a small group of UM patients. However, the findings of this study indicate that a medication, DOX, that was previously disregarded may enter our armamentarium against metastatic UM.

DOX is known to activate Caspase 3 [28]. Knockdown of CREB increased the DOX-induced activation of Caspase 3 (Figures 8 and 9) resulting in an about 70% increase in cell death in both O_2_ conditions, and in an increase in the apoptotic fraction (Table 1). A similar beneficial effect was measured when we combined treatment with DTIC and CREB knockdown.

DOX has a limited therapeutic window due to its toxic effect on normal tissues, with the most adversely affected organ being the heart [29]. We hypothesize that the increase in cell sensitivity to DOX, resulting from CREB knockdown, may result in a decrease in the required active dose of the drug and thus to a reduction in DOX cardiotoxicity.

Latorre *et al.* used DNA and aptamer stabilized gold nanoparticles (GNP) for targeted delivery of anticancer therapeutics. They used GNPs to deliver DOX to cancer cells, including two uveal melanoma cell lines: OMM1.3 and Mel202 [22]. Dong *et al.* used a conjugate of polyethyleneimine (PEI) with DOX via a pH-responsive hydrazone linkage (PEI–Hz–DOX, PHD) and a tumor-targeting folate ligand conjugated to PEI using polyethylene glycol (PEG) as a linker (PEI–PEG–Folate, PPF) in tandem with siRNA targeting vascular endothelial growth factor (VEGF) [30]. They used these nanocomplexes to deliver DOX and the siRNA to breast cancer cells. They found a synergistic effect of the use of DOX and siRNA for VEGF [30]. These results are in agreement with our findings described above. Knockdown of CREB increased the sensitivity to DOX (Table 1 and Figures 8 and 9) and could allow the use of lower concentrations of DOX to avoid its toxicity in patients. Moreover, the unique properties of our system in which the MuLV-based RCR recombinant vectors which generate an ongoing infectious knockdown of CERB in tumor growing cells have specificity to tumor cells more than the above-mentioned approaches of Latorre and Dong.

In this work we demonstrated that the RCR vector expressing shRNA could infect UM cell lines and spread efficiently to knock down the expression of CREB in these cells, resulting in diminished expression of downstream CREB-mediated genes (Figure 4). This knockdown did not affect cell viability under normal growth conditions. However, when infected cells were treated with sub-optimal doses of DOX, the sensitivity to the drug doubled for both cell lines in normoxia. In hypoxia, the sensitivity of Mel270 doubled, and that of OMM2.5 increased dramatically (by 22-fold). Despite the less efficient knockdown of CREB in OMM2.5 vs. Mel270 (Figure 3), OMM2.5 showed a higher dependence on CREB in their response to DOX.

Unlike DOX, DTIC is used in the treatment of metastatic UM, but the efficacy of this drug is far from optimal [31]. In this work, we found that knocking down CREB increases the sensitivity of Mel270 and less so of OMM2.5 to DTIC. This demonstrates the variability in response to chemotherapy of tumor cells from different origins again. The results presented here bring hope that infection with vACE-CREB can increase the sensitivity of some of the tumors to DTIC or DOX and by which, increase the treatment efficacy.

In summary, we have shown that UM can respond to a well-known chemotherapeutic agent (DOX) that was thought to be ineffective for this disease. We have shown that knocking down CREB can increase the sensitivity of some UMs to DOX. The increased sensitivity also to DTIC may indicate that combining CREB knockdown with chemotherapeutic agents may be a general mechanism to improve the sensitivity of solid tumors to chemotherapy.

## MATERIALS AND METHODS

### Cell culture

Human UM OMM2.5 [32] and Mel270 cell lines [33] (verified by STR analysis and a kind gift from Prof. Sarah Coupland, Liverpool, UK) were grown in RPMI supplemented with 10% fetal bovine serum, 2 mM glutamine, 100 IU/ml penicillin, 100 μg/ml streptomycin (Biological Industries) and incubated at 37°C in a humidified atmosphere with 5% CO_2_.

### Plasmids and viruses

The plasmid pACE-GFP (a kind gift from Prof. Noriyuki Kasahara, Los Angeles, California [23]) contains a full-length replication-competent amphotropic MuLV provirus with an additional internal ribosome entry site (IRES)-GFP cassette flanked by BsiWI and NotI restriction enzymes sites. This cassette was replaced by oligonucleotides harboring the H1 promoter driving the transcription of the following shRNA sequences:

5′_GAGAGAGGTCCGTCTAATGTTCAAGAGACATTAGACGGACCTCTCTCTTTTT (pACE-CREB). 5′_ACCAAGATGAAGAGCACCAACCTGAACCATTGGTGCTCTTCATCTTGGTTTTTTT (pACE-NT. Non-target shRNA). See Figure 1 for a schematic presentation of the vectors.

### Virus preparation

HEK293T cells were transiently transfected with either one of the pACE plasmids, described above and in Figure 1, using FuGENE HD reagent (Promega). The medium containing the virus particles was harvested 48h later, filtered (MILLEX-HV, PVDF 0.45μ) and stored at −80°C.

### Viral titer and spread

Virus titer was determined by qPCR (see Results section) comparing RNase P to the viral *env* gene. Virus spread was determined either by flow cytometry of cells infected with vACE-GFP or by qPCR comparing RNase P to viral *env* gene at each time point.

### Immunofluorescence

RCR-infected cells were grown on chamber slides for three days. Slides were fixed with 4% PFA for 2 minutes followed by incubation with pre-cooled 100% methanol for 5 minutes. The slides were washed three times with PBS, blocked with 3% BSA for 30 minutes and incubated for 45 minutes with a primary antibody targeting the p-30 of MuLV driven from R187 cells (ATCC CRL-1912). The slides were washed three times with PBS, incubated with a fluorescent secondary antibody (alexaflour-488 AB-150153 donkey-anti-rat, Abcam) for 45 minutes, and washed three more times with PBS. The nuclei were stained with Hoechst. Stained slides were mounted with Vectashield (Vector Laboratories). Slides were scanned with a Zeiss LSM 710 microscope at x63 magnification and analyzed with ZEN2010 software (Zeiss). Cells were counted for each slide by the number of Hoechst stained nuclei. The ratio of infected cells was calculated for each slide.

### Quantitative real-time PCR

RNA was extracted from the cells using the SV Total RNA Isolation System (Promega), according to the manufacturer's instructions. The purified RNA samples were subjected to reverse transcription using GoScript (Promega), monitored by quantitative 7900HT real-time PCR apparatus (Applied Biosystems) utilizing the GoTaq Real-Time PCR reagents (Promega) and the specific primers: CREB: fp- 5′_CCCAGCACTTCCTACACAGCCTGC, rp5′_CGAGCTGCTTCCUGTTCTTCATTAGACG. The results were normalized to the cellular house-keeping gene GAPDH: fp-5′ CCATCTTCCAGGAGCGAGATCC, rp-5′_GCAAATGAGCCCCAGCTTCTCC.

### Western blot analysis

Western Blot analyzes were carried out by standard procedure [34]. Briefly, equal amounts of total protein were prepared in Laemmli SDS loading buffer, resolved by 10% SDS-PAGE electrophoresis and transferred to PVDF membranes (Millipore). Specific proteins were detected with either CREB, Glut-1 or GAPDH primary antibodies (Abcam) and secondary HRP-conjugated antibody (Promega). The proteins were visualized using the enhanced chemiluminescence (ECL) system (Promega), scanned by the MiniBIS Pro (DNR) scanner and band intensities were quantified by utilizing TINA 20 program (Raytest) on pictures without overexposure of any of the bands.

### Luciferase assay

Cells fully infected with vACE-CREB or vACE NT were seeded in 6-well plates at a concentration of 500,000 cells/well for 24 hours. The infected cells were co-transfected (3μg DNA) with the CRE-mediated luciferase (luc) reporter plasmid vector, pCREluc, together with 0.25 μg of an expression vector expressing the Renilla luciferase gene, phRLSV40, as a transfection control (Promega Corp) using FuGENE HD (Promega Corp). Luciferase activity was determined 48h post-transfection, according to the manufacturer's instructions (Dual-Luciferase reporter assay system, Promega Corp) by an automatic Mithras LB 940 photoluminometer (Berthold Technologies, Oak Ridge, TN, USA). The results were normalized to Renilla luciferase activity.

### Flow cytometry

Stably infected cells with either one of the recombinant RCRs mentioned above were cultivated in normoxic and hypoxic conditions (1%O_2_) with and without a variety of concentrations of either DOX or DTIC for 48h. Cells were subjected to cell cycle analysis by Eclipse- Analyzer.

### Cell viability and activation of Caspase-3

Stably infected cells with either vACE-NT or vACE CREB were cultivated at a concentration of 10,000 cells/well in 96-well plates (4 repeats) at normoxia or hypoxia (0.5% O_2_, in hypoxia boxes) for up to 48 hours. Cell viability and Caspase-3 activity were determined by the Fluorescent Cell Viability and Caspase-Glo 3/7 Assay (Promega) according to the manufacturer's instructions. For the experiments with the chemotherapeutic agents, cells were cultivated with varying concentrations of either DOX or DTIC for 48 hours before determining viability and Caspase-3 activity.

### Statistical analysis

Statistical analysis was performed with JMP 9.0 (SAS). Analysis of variance (ANOVA) was used to compare mRNA and protein levels, survival rates and the Caspase 3 activation levels.

## Abbreviations

UM: uveal melanoma
mUM: metastatic uveal melanoma
DTIC: dacarbazine
DOX: doxorubicin
RCR: replicative competent retroviruses
AMP: adenosine monophosphate
CRE: cyclic AMP-response-element
CREB: CRE binding protein
shRNA: short hairpin RNA
MuLV: Murine leukemia virus
NT: non-target
HCC: hepatocellular carcinoma
GNPs: gold nanoparticles
HIF: hypoxia inducible factor
IRES: internal ribosome entry site
qPCR: quantitative real-time PCR
VEGF: vascular endothelial growth factor
PENK: proenkephalin
